# AIDeveloper: Deep Learning Image Classification in Life Science and Beyond

**DOI:** 10.1002/advs.202003743

**Published:** 2021-03-18

**Authors:** Martin Kräter, Shada Abuhattum, Despina Soteriou, Angela Jacobi, Thomas Krüger, Jochen Guck, Maik Herbig

**Affiliations:** ^1^ Biotechnology Center Center for Molecular and Cellular Bioengineering TU Dresden Dresden 01307 Germany; ^2^ Max Planck Institute for the Science of Light and Max‐Planck‐Zentrum für Physik und Medizin Erlangen 91058 Germany; ^3^ Department of Internal Medicine I University Hospital Carl Gustav Carus TU Dresden Dresden 01307 Germany; ^4^ German Cancer Consortium (DKTK) Partner Site Dresden German Cancer Research Center (DKFZ) Heidelberg 69120 Germany; ^5^ Center for Regenerative Therapies (CRTD) TU Dresden Dresden 01307 Germany

**Keywords:** artificial intelligence, deep neural networks, graphical user interface, image processing, software

## Abstract

Artificial intelligence (AI)‐based image analysis has increased drastically in recent years. However, all applications use individual solutions, highly specialized for a particular task. Here, an easy‐to‐use, adaptable, and open source software, called AIDeveloper (AID) to train neural nets (NN) for image classification without the need for programming is presented. AID provides a variety of NN‐architectures, allowing to apply trained models on new data, obtain performance metrics, and export final models to different formats. AID is benchmarked on large image datasets (CIFAR‐10 and Fashion‐MNIST). Furthermore, models are trained to distinguish areas of differentiated stem cells in images of cell culture. A conventional blood cell count and a blood count obtained using an NN are compared, trained on >1.2 million images, and demonstrated how AID can be used for label‐free classification of B‐ and T‐cells. All models are generated by non‐programmers on generic computers, allowing for an interdisciplinary use.

## Graphical abstract

In this work, AIDeveloper (AID), a software for training neural nets for image classification without need for programming is introduced. AID is open source and executables are provided for easy installation. The authors demonstrate AID by training models for classification of blood cells and natural images as well as detection of differentiated stem cells, reaching state of the art performance.

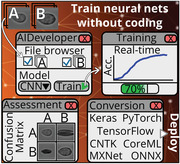

## 1 Introduction

Since the development of the first microscope, progress in life science has become dependent on image acquisition and processing. Over the years, extensive research has led to the development of tools used for quantitative analysis of information in microscopic images. Software such as Cellprofiler,^[^ ^1^ ^]^ Fiji,^[^ ^2^ ^]^ and ImageJ,^[^ ^3^ ^]^ which are widely distributed and used among scientists, allow the user to easily process and quantify features from images. However, quantification using these tools typically results in a set of predefined features that often limit the extent of information that can be extracted from the images. In recent years, the emergence of machine learning (ML) methods, such as deep learning (DL) which uses neural nets (NN), substantially augmented the scope of image processing, quantification, segmentation, and classification. The main advantage of the DL approach is that it does not rely on handcrafted, predefined features, but rather automatically finds a set of optimal features. This can be especially helpful for complex image classification tasks where relevant features are not obvious to the human eye. Cellprofiler 3.0, deepImageJ, and Zen Intellesis are software tools aiming to provide access to DL methods, but lack the ability to train NN. KNIME (https://www.knime.com/) and DL studio (DLS) (https://deepcognition.ai) allow to train NN, but KNIME is not optimized for image analysis and therefore lacks certain functionality such as image augmentation. Both DLS and KNIME, don't allow to adjust hyper‐parameters during training which is available in AIDeveloper (AID).

Recent publications demonstrated the applicability of DL for image‐based object identification in complex biological samples. For example, thrombocyte clusters were identified in human blood samples,^[^ ^4^ ^]^ cell lineage differentiation was predicted during hematopoietic stem cell development,^[^ ^5^ ^]^ skin cancer was classified on dermatologist‐level,^[^ ^6^ ^]^ and mitotic cells were detected in histology images.^[^ ^7^, ^8^ ^]^ The latter showed DL to even outperform histologists in terms of accuracy (accuracy = number of correctly classified images per total number of images). However, only customized, task‐specific algorithms are currently available, as the accessibility and utilization of DL algorithms requires distinct programming skills. Thus, most scientific and clinical applications of DL‐based image classification are restricted to laboratories that can combine expertise in programming and biomedicine. This represents a major drawback, which can only be addressed by the development of DL‐based image processing accessible for the general user.

Here, we present AID (https://www.github.com/maikherbig/AIDeveloper), a flexible ready‐to‐use software to train, evaluate, and utilize NNs for image classification problems available on the platforms Windows, Mac, and Linux with CPU (all platforms) and GPU (Windows only) support out‐of‐the‐box. AID covers the entire workflow of image processing and analysis: from the assembly of datasets and the optimization of NN parameters, to the application of the generated NN to unclassified image sets. A simple user interface allows the user to load different image formats and to visually assess them before and after image size equalization. For training, the user can either choose built‐in NN architectures of different complexities or use custom‐built NNs. In addition, the interface allows the use of pre‐trained models to transfer the learning process, for example, when insufficient training data are available^[^ ^9^ ^]^ or to shorten the training step.

To demonstrate the software´s potential, we trained a convolutional NN (CNN) on CIFAR‐10,^[^ ^10^ ^]^ and Fashion‐MNIST,^[^ ^11^ ^]^ two datasets containing each a collection of images from 10 different classes. We reached a testing accuracy as high as 88% on RGB and 83% on grayscale images for CIFAR‐10 and 93.8% on Fashion‐MNIST. Furthermore, we demonstrate the application of AID for broad biomedical research. First, we trained a model to detect differentiated adipocytes using a relatively small dataset containing only 46 labeled brightfield microscope images. Next, we show the utility of AID for very large datasets by using 1.2 million images of blood cells obtained with real‐time deformability cytometry (RT‐DC),^[^ ^12^ ^]^ in order to generate an automated image‐based whole blood cell count. We trained a model to recognize thrombocytes, lymphocytes, red blood cells, monocytes, neutrophils, and eosinophils based on brightfield images. This is to our knowledge, the first time that a DL algorithm is capable of image‐based classification of major blood cell types from whole blood. Additionally, a live cell image of every cell is available for further analysis. We validated the model by comparing the result to a conventional whole blood count generated by a technique frequently used in clinical practice, which agreed well. Finally, we demonstrate that the tools provided by AID master even challenging classification tasks by training a classifier to distinguish B‐ and T‐cells based on brightfield images from RT‐DC. The resulting model reaches a classification performance that is state‐of‐the‐art for label‐free approaches.^[^ ^13^, ^14^, ^15^ ^]^ Note that the focus of this paper is to introduce the software to the biological community, and demonstrate with a few real‐world biological problems the broad range of its utility for image classification tasks. AID is a ready‐to‐use software package for anyone who wants to start exploring the power of AI‐based image analysis for their own research without the need for any programming skills.

## 2 Results

### 2.1 Using AID to Classify Natural Images from CIFAR‐10 and Fashion‐MNIST

**Figure 1:**
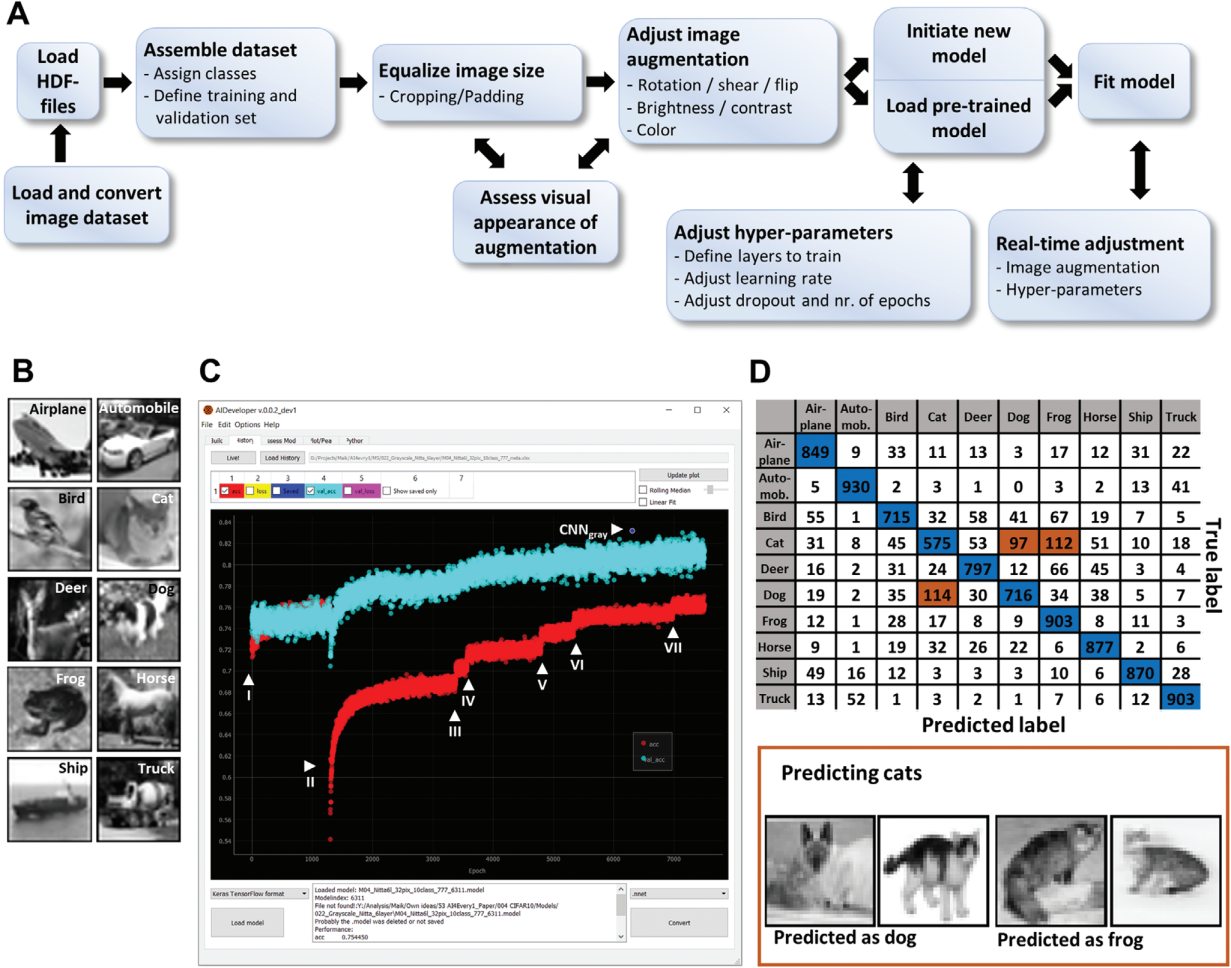
AIDeveloper user interface and workflow. A) A representative workflow of setting up a training process. B) Representative grayscale images of all CIFAR‐10 classes (out of 6000 images per class). C) Screenshot showing the “history”‐tab of AID, which was used to load the training history file of the training process for grayscale images. The scatterplot shows the accuracy (red dots) and the validation accuracy (cyan dots) for each training iteration (also called “epoch”). Arrowheads indicate seven different real‐time user adjustments (I to VII) of image augmentation or hyper‐parameters. CNN_gray_ indicates the model at epoch 6311, which reaches the maximum validation accuracy. D) A confusion matrix indicating the true and the predicted label when classifying the testing set of CIFAR‐10 using CNN_gray_. Matrix items with blue and orange color indicate correctly and incorrectly predicted classes, respectively. Representative images of incorrect predictions from model CNN_gray_ on CIFAR‐10 of class “cat” is shown. The testing accuracy is 81.2%.

AID enables anyone to apply DL for image classification as it guides the user through the entire project pipeline, starting from loading and assembling a dataset, proceeding with training and evaluating a DL model, and ending with classifying new sets of images (**Figure** **1**A; Figure S1, Video S1, Supporting Information). Images for validation and training are simply dragged and dropped into a designated area of the user interface where they are converted to a uniform data format. The validation set is used after every training iteration to validate the generated model. A library of seven different multilayer perceptrons (MLPs) and 23 CNNs of a wide range of complexity is available for choosing a model architecture (Figure S2, Supporting Information). In addition, custom‐built CNNs as well as pre‐trained models are supported. Video S2 explains how custom NN architectures can be defined and added to AIDeveloper. Pre‐trained models can be used either for classifying new sets of images or for re‐purposed training on a different classification task, a technique termed transfer learning.^[^ ^9^ ^]^ Details on the DNN architectures and valid input image dimensions are provided in Table S3, Supporting Information. At the initiation of a training process, AID automatically generates the NN architecture according to the requested input and output dimensions. Image sizes are adjusted either by cropping or padding. AID offers the adjustment of a range of training parameters before and during the training process. These parameters are known as hyper‐parameters and include different image augmentation options, learning rate, and dropout rate (Video S1, Supporting Information). Example images can be visualized before and during the training process in order to assist the adjustment of image augmentation parameters (Video S1, Supporting Information). The accuracy and the validation accuracy are plotted in real‐time after each training iteration. Furthermore, F1 score, precision, recall, support, receiver operating characteristic curve, precision‐recall curve, and further common metrics are shown and can be exported.^[^ ^16^ ^]^ Once a suitable model is obtained (i.e., according to validation accuracy) it can be loaded into AID to assess its performance on testing data (Video S1, Supporting Information). Since developers might want to use a trained model in a different framework, AID also provides conversion tools to protocol buffer format (TensorFlow),^[^ ^17^ ^]^ ONNX, PyTorch, Caffe, MXNet, CNTK, and CoreML (Video S1, Supporting Information). All analyses for this work were performed using AID on a standard consumer PC (Intel Core i7‐3930K @ 3.2 GHz, 32 GB RAM, Nvidia GTX 1080).

To illustrate the software's potential, CIFAR‐10, a common dataset for benchmarking new image classification algorithms, was used. CIFAR‐10 contains 6000 different images of each of the following classes: airplane, automobile, bird, cat, deer, dog, frog, horse, ship, and truck (Figure 1B). 4800 images were used as training set, 200 images of each class as validation set and 1000 images as testing set. We chose a CNN architecture with four convolutional layers (Figure S2N, Supporting Information)^[^ ^4^ ^]^ and converted RGB images to grayscale images to reduce the computational time and accelerate the training process. During training, the image augmentation parameters were adjusted seven times. This caused immediate changes in both the accuracy and the validation accuracy. Importantly, training proceeded with an overall improvement of the validation accuracy, and the best model reached a validation accuracy of 83.2% (indicated as CNN_gray_ in Figure 1C). Furthermore, we trained the same CNN‐architecture using the original RGB images, resulting in a model with a validation accuracy of 87.9% (CNN_RGB_). Both models (CNN_gray_ and CNN_RGB_) were then applied to the testing set. Aided by manual optimization of hyper‐parameters during the training process (Figure 1C), we reached a testing accuracy of 81.2% and 84.7% for CNN_gray_ and CNN_RGB_, respectively. Despite using a fairly low complex CNN with only four convolutional layers CNN_gray_ and CNN_RGB_ would reach place 58 and 50 at https://benchmarks.ai/cifar‐10. The resulting confusion matrix for CNN_gray_ indicates that the distinction between animals, especially classification of cats, dogs, and frogs, was the most erroneous (highlighted in orange in Figure 1D). In AID, confusion matrices are interactive and allow the user to visualize the respective images for each matrix position (Figure 1D bottom panel).

In order to evaluate how trained NN models can be re‐used in AID, we applied a transfer learning approach to optimize the CNN_gray_ model in order to classify images of ten fashion items (Fashion‐MNIST) MNIST^[^ ^11^ ^]^ an image dataset of 10 different classes of fashion items. The dataset contains 7000 images for each class and we used 5800 images for training, 200 for validation and 1000 for testing. The CNN_gray_, previously trained on CIFAR‐10 images (32 × 32 pixels) was further trained on Fashion‐MNIST images (28 × 28 pixel). To allow transfer learning, we utilized the image scaling option in AID to match image sizes. Initially, only the last layer of the pre‐trained CNN was left trainable while all other layers were frozen. By gradually unfreezing all layers^[^ ^18^ ^]^ during training, we reached a robust classification model with an accuracy of 95.1% on the validation set and 93.8% on the testing set. To our knowledge, this is the highest testing accuracy ever reported for Fashion‐MNIST. The training progress and a resulting confusion matrix is visualized in Figure S3, Supporting Information. Furthermore, a video was captured for demonstration (Video S3, Supporting Information). Note that we did not develop a new NN architecture, but used a published architecture^[^ ^4^ ^]^ which we optimized for this classification task.

### 2.2 AID in Life Science and Its Potential for Clinical Diagnostics

AID's ability to train models to natural images could be applied in mobile app development or autonomous driving, where large numbers of natural (everyday‐life) images are encountered.^[^ ^19^ ^]^ Here, we focus on demonstrating its potential for the classification of cell images in life science and clinical diagnostics that also encounter the challenges of processing large image datasets.

**Figure 2:**
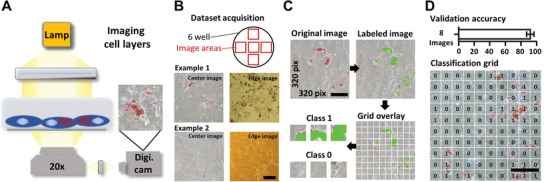
Classifying image tiles containing adipogenic differentiated mesenchymal stromal cells. A) Schematic representation of a light microscope to image cells in 2D culture. Human mesenchymal stromal cells were induced to differentiate into the adipogenic lineage and imaged following Oil Red O staining. B) Image acquisition strategy. Cells were cultured in a six‐well plate and five fixed positions within each well were imaged. Representative images of the center and edge position of two different examples are shown, indicating the variability in image color and staining quality. C) Image‐processing pipeline to obtain training data. Areas of cell differentiation were labeled and the original 320 × 320 pixels images were divided into 100 tiles (32 × 32 pixels). Tiles containing more than 5 labeled pixels were assigned to class 1, others to class 0. D) The bar graph presents the averaged validation accuracy over eight images ± S.D. The image presents the classification of a new image neither contained in the training‐set nor the validation set. The numbers indicate whether a tile was predicted to belong to class 1 (“with differentiation“) or class 0 (”without differentiation”). Scale bars = 50 µm.

Mesenchymal stem cells (MSCs) hold a great potential for the future of cell‐based therapeutic approaches. However, prior to the transplantation it is essential to characterize MSCs and assess their differentiation potential. One classical approach includes MSC differentiation into adipocytes, followed by histological analysis with the lipid dye Oil Red O and manual quantification of the stained cells.^[^ ^20^, ^21^ ^]^ Here, we acquired brightfield images (320 × 320 pixels) of Oil Red O stained adipocytes from different positions in the cell culture well (**Figure** **2**A,B). A trained expert was asked to visually grade and mark the differentiated areas of the acquired images, which varied significantly in brightness, color, and distribution of Oil Red O staining varied significantly (Figure 2B; Figure S4, Supporting Information). We then used the information from the manual labeling and masked the differentiated areas with a uniform green color (RGB: 0, 255, 0) (Figure 2C). Each acquired image was divided into 100 tiles of 32 × 32 pixels and used as a training dataset (Figure 2C). Tiles with more than 5 labeled pixels were assigned to class 1 (“with differentiation”) while all other tiles are assigned to class 0 (“without differentiation”). This translates the image segmentation problem into an image classification problem that can be tackled using AID. We designed a CNN with six convolutional layers, four fully connected layers, and residual connections between layers (Figure S2M, Supporting Information). More complex NNs have the capacity to learn more image characteristics, but as they typically contain more parameters, they tend to overfit.^[^ ^22^ ^]^ This issue can be overcome by increasing the amount of data. Here, due to the limited availability of labeled images, we applied a transfer learning approach, which was shown to reduce the need of data.^[^ ^9^ ^]^ The model was first trained on RGB images from CIFAR‐10 and subsequently optimized for the task of distinguishing tiles with and without differentiation. The final model was validated using eight images, resulting in a validation accuracy of 92.1 ± 4.4% (mean ± standard deviation (S.D.) (Figure 2D; Figure S4, Supporting Information). Finally, we tested the model using an unlabeled image not contained in the training‐ nor validation set. As shown in Figure 2D the tiles classified to class 1 (“with differentiation”) are in good agreement with stained regions.

AID is designed for image classification problems and not for image segmentation. Therefore, the problem had to be converted into a classification problem. A more common practice is to train a model to return a segmentation map of the same size as the input image, allowing for pixel precise predictions.^[^ ^23^, ^24^ ^]^ A software that is designed for image segmentation tasks is ilastik, which we used to train a segmentation model.^[^ ^25^ ^]^ The resulting validation accuracy of 92.2 ± 7.4% shows that the average performance is similar, but as the model failed for some images with pronounced yellowness, the S.D. is higher compared to the model obtained using AID.

**Figure 3:**
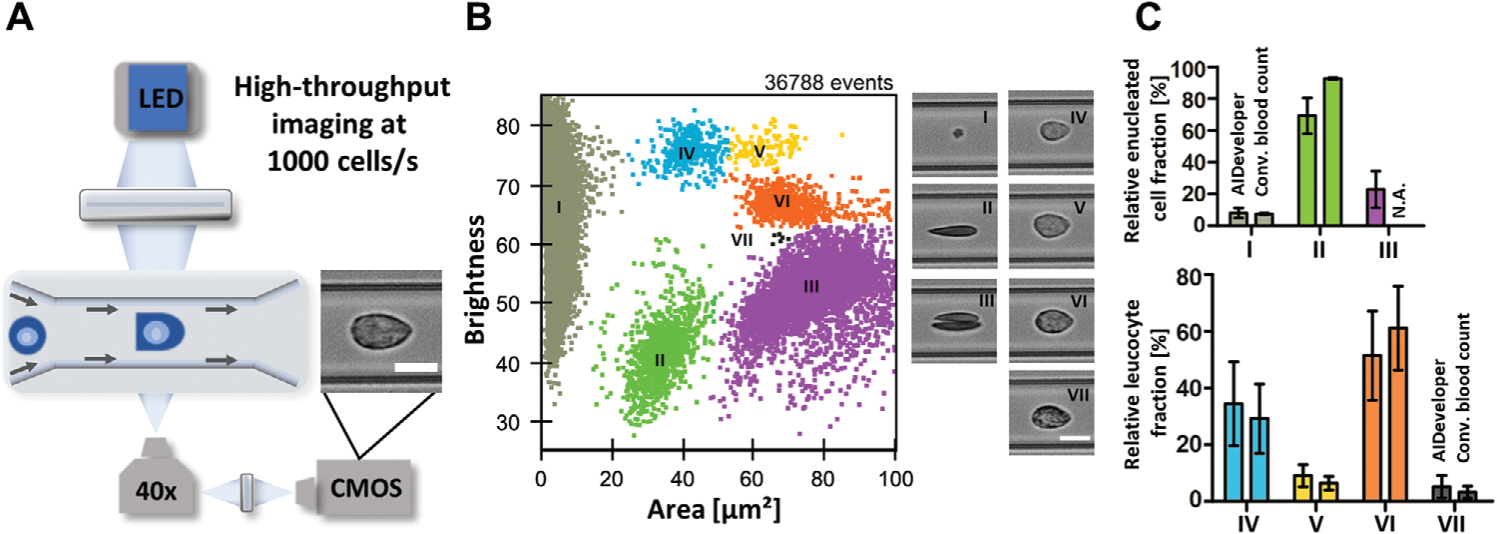
Image‐based whole blood count using RT‐DC and AID. A) Schematic representation of RT‐DC, a high‐throughput imaging technology. A cell suspension is flushed through a channel constriction in a microfluidic chip. Cells are illuminated by an LED and recorded by a high‐speed camera. Multiple parameters including area and average brightness of the cells are determined in real‐time. Scale bar = 10 µm B) The brightness versus area scatter‐plot of whole blood measurements is used to distinguish populations of the major blood cells (I thrombocytes, II erythrocytes, III erythrocyte doublets, IV lymphocytes, V monocytes, VI neutrophils, and VII eosinophils).^[^ ^29^ ^]^ Corresponding images of each population highlight the phenotype of these cells. Manual gating of these populations was carried out to assemble a dataset for training a CNN to perform an image‐based whole blood count. Scale bar = 10 µm C) The bar‐graphs present the relative fraction of enucleated cells (I thrombocytes, II erythrocytes, and III erythrocyte doublets) as well as the leucocytes (IV lymphocytes, V monocytes, VI neutrophils, and VII eosinophils), determined using the CNN and a conventional blood count. Mean ± S.D. of 17 independent blood measurements is displayed.

A broad range of diagnostic techniques relies on high‐throughput imaging of biological samples, followed by manual image‐based analysis by trained experts.^[^ ^26^ ^]^ However, manual analysis of large datasets can delay diagnostics and these images might include useful information that is overlooked by the human eye. An automated AI‐based image classification is potentially the future for advancing the field of image‐based real‐time diagnosis. RT‐DC is an imaging flow cytometer where brightfield images of cells in flow are captured by a high‐speed camera (**Figure** **3**A) at rates of 100–1000 cells per s. In order to increase the frequency of leucocytes, whole blood was first depleted from RBCs by dextran‐sedimentation.^[^ ^27^, ^28^ ^]^ As recently published by Toepfner et al., the individual cell populations can be identified simply by considering cross‐sectional area and brightness, calculated as the average grayscale value of all pixels belonging to the cell (Figure 3B). These parameters are sufficient to distinguish thrombocytes, RBCs, RBC doublets, lymphocytes, monocytes, neutrophils, and eosinophils^[^ ^29^ ^]^ and were used to manually label the dataset. To avoid incorrect labeling, a conservative gating was applied, by excluding events where a distinction was not obvious. Basophils are excluded from our model as they are difficult to distinguish based only on area and brightness. Additionally, datasets were processed by different individuals in order to reduce human bias. Furthermore, they are very rare and we were not able to label a sufficient number of cells for training. We assembled a training and a validation set containing ≈1.2 million images and trained a convolutional NN with 2 convolutional and 3 dense layers (LeNet‐5; Figure S2D, Supporting Information), reaching a validation accuracy of 97.3%. Multiple image augmentation methods were used during training, including changes in orientation (rotation) and brightness levels to ensure robustness of the model. To cope with the relatively high number of RBCs compared to the other cell types, we used the option in AID to randomly sample a defined number of images for each class in each training iteration. This feature would actually be difficult to implement even by direct programming. Testing data were acquired by measuring 17 additional blood samples using RT‐DC and comparing to a conventional blood count measured in parallel under clinical settings. The trained model was applied to classify the images from the RT‐DC experiments. The resulting cell count was comparable to the conventional blood count (Figure 3C). Using RT‐DC, an additional population of RBC doublets was found, which is not reflected in the conventional blood count.

**Figure 4:**
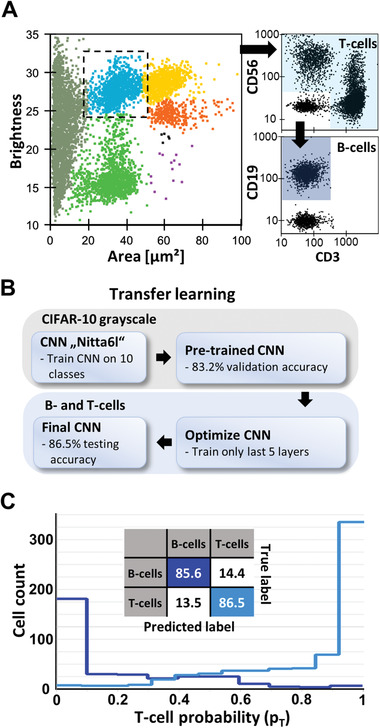
Label‐free classification of B‐ and T‐cells from human blood. A) Gating strategy for acquiring training data for B‐ and T‐cell classification. A scatter‐plot (brightness vs area) of human fractionated blood, measured using real‐time deformability and fluorescence cytometry (RT‐FDC) is shown. Lymphocytes were gated (dashed square) based on brightness and area.^[^ ^29^ ^]^ B‐ and T‐cells were labeled according to standard surface CD markers (CD3—T‐cells, CD19—B‐cells, and CD56—NK‐cells). B) A representative schema of a transfer learning process, which can be easily applied in AID. The pre‐trained CNN_gray_, with a validation accuracy of 83.2% on the CIFAR‐10 dataset, was loaded into AID and optimized to classify images of B‐ and T‐cells, acquired from fractionated blood using RT‐FDC. A final validation accuracy of 89.3% and a testing accuracy of 86.2% was achieved. C) Confusion matrix of B‐ versus T‐cells as well as the probability histogram showing the performance of the model on the testing set. The abscissa in the histogram shows the predicted probability to be a T‐cell (*p*_T_).

To further emphasize the potential of DL and AID in the context of blood analysis, we set out to classify lymphocytes into B‐ and T‐cells label‐free, a task not yet feasible routinely in clinical diagnostics. To discriminate between B‐ and T‐cells in whole blood, we used real‐time fluorescence and deformability cytometry (RT‐FDC).^[^ ^30^ ^]^ This technique is similar to RT‐DC, but allows for fluorescence detection in parallel to imaging. We generated a labeled dataset from three healthy donors, by using a panel of three fluorescent antibodies specific for each cell type; “cluster of differentiation” 19 (CD19) for B‐cells; CD3 for T‐cells and CD56 for natural killer (NK)‐cells, a subset of T‐cells. As previously described, we identified lymphocytes based on area and brightness (blue in **Figure** **4**A). B‐ and T‐cells were identified based on expression of the different fluorescent markers as shown in Figure 4A. We used this ground truth to assemble a dataset which can be used to train a model to distinguish B versus T lymphocytes using the bright field images only. Training and validation sets were assembled using data from three donors. We used a 4th measurement from a different healthy donor as testing dataset. Acquired images for B‐cells (CD19^+^ events) and T‐cells (CD19^+^ or CD56^+^ events) were loaded into AID and assigned to individual classes. We used a transfer learning approach by loading the previously trained CNN_gray_ (Figure 1C; Figure S2N, Supporting Information), into AID and continued training using RT‐FDC images of B‐ and T‐cells (Figure 4B). While CNN_gray_ was trained on a very different image dataset, elementary image features such as edges or corners also exist. Such simple image features are described by the first convolutional layer^[^ ^31^ ^]^ so we omitted this layer from training to reduce the risk of overfitting and the computation time. We continued training over the course of one month to promote a model with highest validation accuracy possible, demonstrating stable execution of AID for long run‐times. The final model reached a validation accuracy of 89.3%. We used AID to apply the model on testing data and obtained the following scores: testing accuracy = 86.2%, F1 score = 89.3%, precision = 92.3%, recall = 86.5%, and an area under curve of the receiver‐operating characteristic and the precision recall curve of 94% and 97%, respectively. The probability histogram of the testing‐set (Figure 4C) indicates that above a threshold of *p*_T_ = 0.9, 98.3% of the events are classified as T‐cells and below *p*_T_ = 0.1, 95.9% of the events as B‐cells.

## 3 Discussion

### 3.1 Graphical User Interface Provides Simple Access to Deep Learning Methods

Advances in the acquisition of imaging data necessitate the availability and accessibility of automated processing tools that can drive scientific insights toward routine application. The exponential rise seen in the last decade in artificial intelligence publications^[^ ^32^, ^33^ ^]^ was accompanied with a rise in DL models for addressing scientific problems; such as medical image processing, diagnostics, image interpretation, and classification. However, despite the availability of multiple imaging software that employ DL approaches, a ready‐to‐use software is to our knowledge not yet available. Moreover, the fact remains that real‐life application of DL and training of NNs require expert programming skills that pose a real challenge to non‐expert users. The open source community is capable to drive such software development, but the continuous evolution of programming environments and the fast turnover of software libraries impedes a cooperative progress. More importantly, the complex terminology of DL can prevent correct data assembly, successive training, and application of NNs, especially for users with limited ML knowledge. Furthermore, even installing intercompatible software requirements for DL can be a substantial burden, especially, when GPU support is desired.

### 3.2 Comparison to Other Machine Learning Software

AID was designed to assist researchers to tackle image classification problems, without the complexity of expert coding skills or installation of a programming environment. Advances in automated image data acquisition has led to the development of various software that offer image analysis using DL architecture. Commercial software, though easy to use, are limited by their cost, and more importantly by the limited accessibility on how the data are processed. To address these issues, the open source community has developed many tools that offer researchers more flexibility and ease‐of‐use, but also allow image analysis using DL architectures.

We compared AID to eight other existing open‐source and commercial software and the major findings are summarized in Table S2, Supporting Information. For example, Cell Profiler 3.0, deepImageJ, and ilastik, are open‐source software with a user‐friendly interface that allow the user to employ pre‐trained NN for image segmentation and feature extraction.^[^ ^25^, ^34^, ^35^, ^36^ ^]^ In ilastik, the extracted features can then be used to train a random forest^[^ ^37^ ^]^ for image segmentation. A drawback of ilastik is the requirement for additional packages such as Python, Python libraries, and CUDA (optional for GPU support) in order to use the pre‐trained NN. Zen Intellesis, is another image segmentation tool that employs pre‐trained CNNs for feature extraction to train random forests, but unlike ilastik, it does not require installation of auxiliary software. However, the program is only commercially available.

Overall many existing graphical user interface (GUI) based tools that apply ML to classify or segment images, including Cell Profiler 3.0, deepImageJ, and ilastik, lack the option to train DNN. DIGITS by Nvidia, offers the possibility to train DNNs but requires installation of multiple Python packages and for GPU utilization, installation of CUDA is required. In addition DIGITS provides limited access to hyper‐parameters and a limited number of NN that require specific input image dimensions. To adjust a model to other image dimensions or numbers of classes, programming skills are required. DLS from DeepCognition (https://deepcognition.ai) is a proprietary all‐round software solution for training DNNs. Similar to AID, image augmentation methods are readily implemented. Overall, DLS is a very complete and powerful software. Unfortunately, offline execution of DLS and GPU acceleration are only supported in a paid version. KNIME (https://www.knime.com) is an open source software with user interface that offers multiple ML techniques, including DNNs. KNIME can be applied to many data formats, including images, however as it is not specialized in image analysis, it lacks image augmentation methods. In addition, in order use DL methods in KNIME, a Python distribution and particular packages have to be installed manually and for GPU support further installations (e.g., CUDA) are required. Finally, both DLS and KNIME, do not allow adjustment of hyper‐parameters during training which is available in AID. However, while KNIME and DLS allow processing of multiple data types, AID is limited to 2D squared images. For a detailed comparison of AID with numerous existing ML software tools please refer to Table S2, Supporting Information.

AID addresses these issues by providing a standalone executable, including an intuitive GUI, which allows also non‐programmers to train, evaluate, and apply NNs to their image datasets. Moreover, AID includes different NN architectures with different levels of complexity ranging from very simple MLPs to contemporary CNNs with many layers. All these networks can be extended, updated, or replaced easily. The DNNs can be selected in a dropdown list where the models appear in order (low complexity first). Hence, users could initially train a low complexity NN (top in the list) and subsequently choose to train a more complex DNN (lower in the list) if a higher accuracy is desired. Moreover, the user‐interface of AID offers the option to omit layers from training. This strategy reduces the risk of overfitting and lowers the computational time since less parameters need to be updated during training. Larger NNs might be favorable to maximize the classification accuracy while smaller models allow for real‐time applications. Facilitating AID, label‐free cell sorting for neutrophils from human blood was already demonstrated.^[^ ^38^ ^]^

AID guides the user through the workflow to develop classification models, quantify their performance, and apply them to new data. Established methods for image normalization and augmentation are integrated. Interactive visualization tools allow a seamless link between user‐settings and their effect on image data or the training process. For analysis of unbalanced datasets (i.e., different numbers of images in each class), AID provides tools to randomly sample a defined number of images in each training iteration—a feature not straight‐forward to implement by programming (Figure S5A, Supporting Information). Furthermore, the loss contribution of the classes can be balanced using scalar coefficients (Figure S5B, Supporting Information). The software assists the user in choosing sensible coefficients. Automatic documentation of the training process keeps track of all user‐settings and model performance progression during training. Standard methods for quantification of model performance are embedded. Tooltip annotations help the user to understand the underlying ML concepts and roles of different hyper parameter settings. Execution of AID is identical on Windows, Mac, and Linux, allowing for reproducibility, sharing of models, and continuation of training using different PC setups. During creation of the standalone executable, we focused on achieving compatibility to a broad range of PC systems. Therefore, we integrated support for CPU, single GPU, and multiple GPUs (GPU support is currently only available for Windows OS). Dedicated GUI elements allow to select the hardware for training. Currently, any Nvidia graphics card with a compute capability larger 3.7 is automatically detected. As AID is open source it can also be run from script which allows more broad GPU support. Thus, AID could empower people to use DL for image classification, with implications for a wide range of disciplines, from life science to app development.

### 3.3 Introducing AID Using CIFAR‐10 and Fashion‐MNIST

We introduced the features of AID using CIFAR‐10, a dataset of images commonly used as a standard to compare and benchmark image classification methods. Overall, AID can be used to load published NN architectures and train new models to classify both RGB and grayscale images. AID can convert RGB into grayscale upon user request. Furthermore, when creating a new NN, AID adjusts the input layer of the model according to the channel dimensions and user‐defined input image size. Here, image input size is adjusted by center‐cropping or padding. Currently, only squared input images are supported, but in the future rectangular image support will be implemented. Moreover, an interactive confusion matrix is displayed when a model is applied to new data (e.g., testing data), allowing the user to visualize correctly or incorrectly classified images.

The models for CIFAR‐10 (CNN_gray_ and CNN_RGB_) were trained on an Intel Core i7‐3930K, which took 77.9, and 210.4 h, respectively. Training times of multiple hours are typical in the field of DL. In another study, which achieved an accuracy of 98.52% for CIFAR‐10, training times of 5000 GPU hours (estimated for an Nvidia Tesla P10) are reported.^[^ ^39^ ^]^ Using more efficient hardware, the training time can be lowered. For example by switching from CPU (Intel Core i7‐3930K) to GPU (Nvidia GTX 1080), we achieved a speedup of ≈20× when training the model for Fashion‐MNIST, which allowed us to obtain a record breaking testing accuracy of 93.8% within only 3.2 h training time. A benchmark dashboard (http://fashion‐mnist.s3‐website.eu‐central‐1.amazonaws.com/#) summarizes other approaches to Fashion‐MNIST and training times up to 50 h are reported.

### 3.4 AID for Life Science Applications and Clinical Blood Diagnostics

Biomedical research and clinical diagnostics often utilize image‐based techniques, such as microscopy, flow cytometry, histopathology, and immunohistochemisty, which result in an increasing demand for automated image classification. While large tech‐companies such as Google (https://deepmind.com/about/health) and Microsoft (https://www.microsoft.com/en‐us/research/project/medical‐image‐analysis) have initiated dedicated projects that focus on medical imaging, smaller research groups or start‐up companies cannot always afford specialized personnel for data analysis. Furthermore, enabling experts in a respective discipline to independently perform image analysis is advantageous for accurate data interpretation. AID could address a variety of biological applications including classification of histopathology images or identification of novel subpopulations within a larger dataset of imaging flow cytometry, as well as quantification of immunofluorescent and immunocytochemistry images. Here, we chose three applications to demonstrate the potential use of AID in biomedical research and/or as a tool for facilitating clinical diagnostics: 1) quantification of adipogenic differentiation of MSCs from brightfield microscopy; 2) label‐free blood count; and 3) label‐free B‐ versus T‐cell discrimination from imaging flow cytometry (RT‐DC).

#### 3.4.1 Quantification of Adipogenic Differentiation of Mesenchymal Stem Cells

MSCs are a major source of stem cells used in cell therapy.^[^ ^40^ ^]^ Their differentiation potential is assessed by measuring areas of adipogenic differentiation in an MSC layer, which involves cell staining, imaging, and classification of the differentiated areas. Manual classification is laborious and time consuming. Currently, computer‐based quantification is challenging because of variability in image brightness, cell density, uniformity of staining, and morphological differences (Figure 2B; Figure S4, Supporting Information). For example, ilastik, reported that classifier performance can be affected by imaging conditions, such as intensity or imaging artefacts.^[^ ^25^ ^]^ Our segmentation model trained using ilastik reflects such issues as the model fails for certain images with pronounced yellowness. Implementing additional color normalization methods could help to improve the classification accuracy. On the contrary, our validation results confirm that the model trained using AID can robustly quantify MSC differentiation despite different imaging artefacts and changes in image intensity. Ultimately we believe that using AID for the analysis of microscopy images or histopathology samples has the potential to reduce the burden on the personnel and also reduce delays in diagnosis.

We used a transfer learning approach to train a NN with CIFAR‐10 images and then continued training with images of differentiated MSCs; achieving a validation accuracy of 92.1%. In fact, application of this model to a classical research quantification task revealed that the model returned sensible predictions for new data.^[^ ^41^ ^]^

Datasets were acquired using one imaging system and the resulting classification model is optimized for the corresponding image phenotype. Before employing the model for data from a different imaging system, an assessment of the classification performance is necessary.

The computational time required for classification (inference time) is ≈1.7 ms for a single tile (32 × 32 pixels) and 0.2 s for a complete image (320 × 320 pixels) on an Intel Core i7‐3930K CPU, rendering this model applicable for high‐throughput analysis. In comparison, manual labeling takes ≈20 s per image, which is 100 times longer. The DNN was trained using AID on an Intel Core i7‐3930K, which took 28.2 h.

#### 3.4.2 Label‐Free Blood Count

The complete blood count is a routine diagnostic tool used to assess the health of a patient and detect abnormalities such as infections or blood disorder. We have previously shown that utilizing RT‐DC we can identify all major blood cell types based on their morpho‐rheological phenotype.^[^ ^29^ ^]^ Whereas manual microscopy can capture ≈1 image per second, imaging cytometers, like RT‐DC, easily speed up this task by a factor of 1000, resulting in considerably larger datasets. Here, we highlight the ability of AID to train CNNs on large datasets, by employing RT‐DC to capture 1.2 million brightfield images of blood cells (Figure 3A). Cell subpopulations were gated manually, according to size and brightness (Figure 3B; as described in Toepfner et al., 2018) prior to loading into AID and performing training. Despite the simple dimensionality of the gating strategy, an automated classification strategy would not be feasible, since the brightness in RT‐DC is manually adjusted by the user for each experimental setup. These variations between experiments can substantially affect the performance of an automated classification strategy. However, AID has proven to achieve high classification performance despite these technical alterations. Indeed the resulting blood count was comparable to a conventional clinical blood count (Figure 3C) indicating that the model is not influenced by mislabeled cells. This was expected since DL algorithms were shown to be robust against labeling noise, especially when using large datasets.^[^ ^42^ ^]^

Another advantage of this model is that the inference time for a single image is ≈1 ms (on an Intel Core i7‐3930K CPU), corresponding to a prediction rate of 1000 cells per s, which matches the image acquisition rate of RT‐DC. Thus, in principle the model can be applied for on‐the‐fly prediction. Moreover, AID was able to detect a subpopulation of RBC doublets that is not readily detectable by standard diagnostics techniques. This could complement the conventional whole blood count and provide additional information that might facilitate the pathologists to speed up and improve diagnosis. For example, RBC aggregates could be used as a diagnostic marker since their appearance is correlated to infection through increased fibrinogen concentration.^[^ ^43^, ^44^ ^]^ Furthermore, RBC aggregation is linked to erythrocyte sedimentation rate, which is a widely used marker to diagnose inflammatory or pathophysiological conditions.^[^ ^45^ ^]^ The DNN was trained using AID on an Intel Core i7‐3930K, which took 6.8 h.

#### 3.4.3 Label‐free Discrimination of B‐ and T‐cells

Lymphocytes, which includes B‐, T‐ and NK‐ cells, are a subset of blood cells that are involved in immune responses. In clinical diagnostics, lymphocyte cell counts are critical for determining whether a patient's immune system is fighting an infection. Conventional B‐ and T‐ cell counts require fluorescent labeling and flow cytometry analysis, as these cells are morphologically indistinguishable.^[^ ^46^ ^]^ Here, we demonstrated that we were able to train a CNN to distinguish two subtypes of lymphocytes, B and T‐ cells, using only brightfield images obtained from RT‐DC. Our results show that the classification performance of that model is at least similar to other publications showing label‐free discrimination of B‐ and T‐cells.^[^ ^13^, ^14^, ^15^ ^]^ These results suggest feasibility of label‐free image‐based discrimination of subpopulations of cells, which could be used to complement blood cell counts. Moreover, one promising prospect of AID is to apply it for the detection and isolation of cells based on their morphological properties. Given the large margin between the predicted probabilities of B‐ and T‐cells (Figure 4C), combining label‐free image‐based sorting using this model could result in highly pure B‐ or T‐cells samples. Label‐free image‐based sorting was recently demonstrated for neutrophils using AID and platelet aggregates.^[^ ^4^, ^38^ ^]^ This sorting approach opens the possibility to use the cells for downstream applications without the risk of contamination from molecular labeling, and also reducing costs and preparation time. The DNN for B versus T cell discrimination was trained using AID on an Intel Core i7‐3930K, which took 720.0 h. We deliberately used the CPU to demonstrate stable execution of AID for such long training times. In practice, we would recommend using a GPU for this training task. Using, for example, an Nvidia GTX 1080 we achieve approximately a 20× speedup on our setup, which would translate to a drop of the training time to 36 h.

In conclusion, we present a software tool which drastically increases the accessibility of DL‐based image classification to non‐experts. AID can handle RGB and grayscale images in small and large datasets and has state‐of‐the‐art techniques for improved training of NNs, such as image augmentation and transfer learning already implemented. Moreover, since AID can employ CPU, GPU, or even multiple GPUs for processing, it can run on almost any PC system; without the need for additional, expensive hardware. In a proof of concept approach, we have demonstrated the power of AID for obtaining robust classifiers for multiple use‐cases covering a wide spectrum of applications. Further work is required to evaluate the performance of AID in other biomedical areas including immunohistochemistry, radiology, histopathology, and other medical imaging techniques. We envision that AID can be applied by anyone, ranging from expert programmers to clinicians, to image classification problems in life science and beyond; with the potential to improve accuracy and speed of interpretations.

## 4 Experimental Section

##### Software Development

AIDeveloper (AID) is an open source software (license BSD 3‐Clause License; https://github.com/maikherbig/AIDeveloper), written in Python 3.5 using PyQt (package for GUI), Keras (https://keras.io/) and TensorFlow^[^ ^17^ ^]^ (packages for DL), and further open source Python packages (Table S1, Supporting Information). AID features CUDA (Nvidia) out‐of‐the‐box (Windows only), allowing for automatic detection of Nvidia GPUs (compute capability > 3.7). Dedicated UI elements allow to distribute processing tasks to CPU, GPU, or multiple GPUs.

PyInstaller (https://www.pyinstaller.org) was used to generate standalone executables of AID for Windows, Mac, and Linux. No installation of Python or CUDA was required when running AID from these executables. Alternatively, a detailed protocol is also provided to set up a Python development environment for AID including all required packages (Video S4, Supporting Information). Within such an environment, developers could implement new features for their specific task and even create new standalone executables (Video S4, Supporting Information). The novelty of AID lays in the simple access to powerful image classification algorithms through an intuitive user interface. AID addresses the issue of a growing number of image datasets which requires fast and reproducible analyses. During a training process, AID automatically documents which data were used and tracks all hyper‐parameters. Hence, training routines in AID are transparent and repeatable.

AID supports 2D grayscale and RGB images and provides tools for conversion. To convert RGB images to grayscale, AID uses the luminosity method, which performs a weighted average of the channels of an RGB image, accounting for the higher sensitivity of the human eye to green color: gray = 0.21 × R + 0.72 × G × 0.07 × B (Figure 1B). To convert grayscale to RBG, AID stacks three copies of the grayscale image.

Optionally, images can be scaled which is useful for example when working with multiple image sets that were captured at different magnification. Beside nearest neighbor interpolation, AID also provides linear, quadratic, and cubic interpolation.^[^ ^47^ ^]^ The final size of the images is set by the user and AID performs center cropping or padding to obtain the requested size. The final cropping step is performed after random rotation of images in order to avoid edge effects. Further image augmentation options are available and choosing sensible parameters is assisted by the display of example images. As image augmentation is computationally expensive, AID provides efficient algorithms leveraging implementations of OpenCV.^[^ ^48^ ^]^

Image normalization methods improve training speed. AID provides the option to divide each pixel value by 255. Furthermore, two standard scaling methods are implemented, which use the mean and S.D. of the whole training set or of each image individually.

By default, the entire dataset were loaded into RAM before a training process. Alternatively, loading to RAM could be omitted and datasets larger than the available RAM memory could be processed from hard disk.

NN selectable in AID are defined in a Python script (model_zoo.py), which also contains minimal examples. By editing this script, the user can modify and add NN architectures which are then available within AID. Editing the model_zoo.py does not require an installation of Python but can be accomplished using a text editor (Video S2, Supporting Information).

For training NN, a PC system with an Intel Core i7‐3930K CPU @ 3.2 GHz, an Nvidia GTX 1080 GPU, and 32 GB RAM was used.

##### CIFAR‐10 and Fashion‐MNIST

CIFAR‐10 is a labeled image dataset containing 60 000 RGB‐images of 10 different classes.^[^ ^10^ ^]^ The dataset were downloaded from https://pjreddie.com/projects/cifar‐10‐dataset‐mirror. In this dataset, 50 000 images are dedicated to training and 10 000 to testing. 200 random training images of each class were picked to create a validation set.

During training, facilitating a CNN with 4 convolutional and 2 dense layers (nitta_6layer; Figure S2N, Supporting Information) on grayscale images, the hyper‐parameters were adjusted seven times and changes were tracked automatically in a meta‐file. For normalization, the pixel values were divided by 255. Images were randomly flipped along the central vertical axis and randomly rotated by up to ±5°. Furthermore, images were shifted in each direction by up to one pixel and zoomed by a factor of up to ±0.35. The brightness of the images was altered after each training iteration by randomly adding ±15 and multiplying the grayscale values by a random number between 0.6 and 1.4 (before normalization). Gaussian noise with random scale of up to 10 was added.

Fashion‐MNIST is a labeled image dataset containing 7000 images for each class of 10 classes of fashion items. 5800 images were used for training, 200 for validation, and 1000 images for testing. The Fashion‐MNIST grayscale images were 28 × 28 pixel. The dataset were downloaded from https://github.com/zalandoresearch/fashion‐mnist. The image size was increased to 32 × 32 pixels by scaling using a factor of 1.15 (nearest neighbor) in order to allow for transfer learning using CNN_gray_. After loading CNN_gray_, all layers but the last one were frozen (i.e., no updates of weights during training). Each time, a plateau in validation accuracy was reached, a further layer was made available for training. Images were randomly flipped along the vertical axis, rotated by ±10°, shifted in each direction by up to one pixel. Brightness levels were randomly altered by adding up to ±15 and multiplying each image by values between 0.7 and 1.3. Gaussian noise with random scale of up to 5 was added.

##### Mesenchymal Stromal Cell Isolation, Culture, Differentiation, and Dataset Acquisition

MSC isolation was performed in compliance with the Declaration of Helsinki. Bone marrow (BM) aspirates were taken from healthy volunteer donors while routine BM donation, after obtaining informed written consent, and cells were isolated (ethical approval no. EK221102004, EK47022007) by adapting a previously reported method.^[^ ^20^ ^]^ In detail, BM aspiration was done according to the rules of the BM transplantation center of the medical faculty of the TU Dresden (Universitätsklinikum Carl Gustav Carus Dresden). After BM transplantation the remaining BM was washed out from the transplantation pouch by adding phosphate buffered saline (PBS; Sigma Aldrich, Germany) at a ratio of 1:2. BM/PBS mixture was removed from the pouch using a 5 mL syringe (VWR International, USA) and a blunt filling needle (Becton Dickinson, USA). A density gradient centrifugation (400 g for 15 min at room temperature, slowest acceleration, and no brake setting) was performed using a 20 mL BM/PBS aliquot layered over 12.5 mL of a 1.073 g mL^−1^ Percoll solution (Biochrom GmbH, Germany) in a 50 mL conical centrifuge tube (Greiner Bio One, Germany). The fraction of mononucleated cells (MNCs) was transferred to a T‐25 cell culture flask (Greiner Bio One, Germany) in 10 mL MSC medium and allowed to adhere for 24 h. MSC medium was prepared using Dulbecco's modified Eagle medium (DMEM) with glucose and L‐glutamine (DMEM Culture Media; VWR, USA) and 10% fetal calf serum (BD, USA). After 24 h MSCs were purified by removing medium supernatant from the cell culture flask and excess cells (non‐adherent) were washed of by 3 washing steps using 15 mL pre‐warmed PBS. For adipogenesis, MSCs were detached by a washing step with 15 mL PBS, followed by a 5 min incubation time using 2 mL trypsin‐EDTA (0.25%) (Thermo Fisher, USA). Trypsin was blocked by adding 5 mL MSC medium and 10^5^ MSCs were transferred to 3 wells of a 6‐well tissue culture plate (Greiner Bio One, Germany) in 2 mL per well MSC medium. Adipogenic differentiation was induced when cells reached 80% confluency as previously described.^[^ ^21^ ^]^ Briefly, adipogenesis was induced by exchanging MSC culture medium with a medium containing 1 µmol L^−1^ dexamethasone, 0.5 mmol L^−1^ 3‐isobutyl‐1‐methylxanthine, 100 µmol L^−1^ indomethacin, and 10 µmol L^−1^ insulin (Sigma Aldrich, St. Louis, USA) in MSC culture medium for 14 days. All cultures were kept at 37 °C with 5% CO_2_ in a water‐jacked incubator. Medium changes were performed weekly. For histological visualization differentiated cells were fixed with 4% paraformaldehyde (Merck KGaA, Darmstadt, Germany) in PBS. Adipogenic differentiation was assessed by 2 to 5 min Oil Red O staining with 0.1% Oil Red O solution (Sigma Aldrich, St. Louis, USA) in ethanol (VWR International, USA), followed by five washes with distilled water. To generate the image dataset an inverted microscope (Axiovert 25, Carl‐Zeiss, Jena, Germany) equipped with a digital camera (Olympus E330, Olympus, Hamburg, Germany) was used. Images from five different positions of each well were taken (Figure 2A,B).

Labeling was performed by an expert marking each pixel that corresponded to an area of differentiated MSCs, resulting in a pixel precise map. In total, 46 images from 16 different donors were labeled. 38 labeled images were used for training and the remaining 8 images for validation. Each original image of 320 × 320 pixels in size was partitioned into 100 tiles of 32 × 32 pixels (Figure 2C). The chosen size of 32 × 32 pixels approximately met the size of differentiated area and also reflects a compromise between large tiles which would often contain several differentiated areas and single‐pixel‐tiles that would prevent a model from learning about the morphology of the differentiated areas. A tile was assigned to class 0 (“without differentiation”), if it contained less than five marked pixels or to class 1 (“with differentiation”) if it contained more or equal to 5 pixels. After equally partitioning all images, tiles of class 1 were clearly under‐represented. Therefore, more tiles from random locations containing more than four marked pixels were added. This strategy helped to balance the dataset and allowed to obtain tiles with objects at various locations in the image. The latter could help to train a more translation invariant model. A single image of a completely different dataset was used to test the trained model (Figure 2D) in order to highlight the applicability of the obtained model to new input.

The random forest based segmentation model was trained using ilastik v.1.3.3 using the following features: Gaussian smoothing, Laplacian of Gaussian, Gaussian gradient magnitude, difference of Gaussians, structure tensor eigenvalues, and Hessian of Gaussian eigenvalues.

##### Real‐time Fluorescence and Deformability Cytometry for Blood

RT‐DC and RT_FDC were performed as described elsewhere.^[^ ^12^, ^30^ ^]^ Briefly, a microfluidic chip made from polydimethylsiloxane (SYLGARD, Dow Corning, USA) was mounted on an inverted microscope (Observer Z1, Zeiss, Jena, Germany) equipped with an LED (CBT‐120, Luminus Devices, USA) and a high‐speed camera (EoSens CL MC1362, Mikrotron, Germany) (Figure 3A). Two syringe pumps (NemeSyS, Cetoni, Germany) were used to deliver cells suspended in measurement Buffer (MB) and sheath fluid into the chip at a sample flow rate of 0.015 µL s^−1^ and a sheath flow rate of 0.045 µL s^−1^ resulting in a total flow rate of 0.06 µL s^−1^. For suspending cells, a MB based on Mg^2+^‐and Ca^2+^‐free PBS (Sigma Aldrich, Germany), supplemented with 0.6% w/w methylcellulose (Sigma Aldrich, Germany) viscosity adjusted to 26 mPa s at room temperature, was used. An image of every cell was captured in a region of interest of 250 × 80 pixels at a frame rate of 2000 fps inside a constriction channel of 20 µm × 20 µm cross‐section (Figure 3A). RT‐DC technology, RT‐FDC technology and all consumables are commercially available (Zellmechanik Dresden GmbH).

For preparation of whole blood samples, venous blood was drawn from human donors using a 20‐gauge multifly needle (Sarstedt, Germany) into sodium citrate tubes (S‐Monovette 10 mL 9NC, Sarstedt, Germany) by vacuum aspiration. Whole blood samples were prepared by diluting 50 µL of whole blood in 950 µL of MB as previously published.^[^ ^29^ ^]^

To prepare RBC‐depleted blood samples, 2 mL of a 6% dextran solution (Dextran T500, Pharmacosmos A/S, Denmark) diluted in sodium chloride (0.9% Sodium Chloride Irrigation, Baxter Healthcare, Switzerland) were added to 10 mL of whole citrated blood drawn using a 20‐gauge multifly needle (Sarstedt, Germany) into sodium citrate tubes (S‐Monovette 10 mL 9NC, Sarstedt, Germany) by vacuum aspiration. After gentle mixing, RBCs were allowed to sediment for 30 min.^[^ ^27^, ^28^ ^]^ The supernatant was transferred to a 15 mL conical centrifuge tube (Greiner Bio One, Germany) and centrifuged for 10 min at 120 g (Universal 30RF, Hettich, Switzerland). After removing the cell‐free plasma, the pellet was resuspended in 2 mL MB.

For B‐ and T‐cell classification fractionated blood was used. Blood aspirates were diluted in PBS (Sigma Aldrich, Germany) at a ratio of 1:5, followed by a density gradient centrifugation (400 g for 15 min at room temperature, slowest acceleration and no brake setting) using a 20 mL aliquot layered over 12.5 mL of a 1,073 g mL^−1^ Percoll solution (Biochrom, Berlin, Germany) in a 50 mL conical centrifuge tube (Greiner Bio One, Germany). The fraction of MNCs was transferred to a 15 mL conical centrifuge tube (Greiner Bio One, Germany) and the tube was filled up with PBS (Sigma Aldrich, Germany) to 15 mL. The cells were centrifuged (400 g, 5 min at room temperature) and resuspended in PBS to a concentration of 5 × 10^6^ cells mL^−1^. 100 µL aliquots were used to stain for B‐cells using 5 µL of an antibody against CD19 (coupled to allophycocyanin; APC) (Clone: REA675; Miltenyi Biotec, Germany), T‐cells using 5 µL of an anti‐CD3 antibody (coupled to fluorescein; FITC) (Clone: REA613; Miltenyi Biotec, Germany), and NK‐cells, as a subset of T‐cells using 5 µL of an anti‐CD56 antibody (coupled to phycoerythrin; PE) (Clone: REA196; Miltenyi Biotec, Germany). After another washing step using PBS, and a centrifugation step (400 g, 5 min at room temperature) the cells were finally resuspended in MB. Each blood sample was stained and measured independently. Manual gating was performed for each measurement independently and by different individuals to reduce human bias. For each replicate, the individual subpopulations appear at slightly different fluorescence intensities, but were always clearly distinguishable (see Figure 4A).

RT‐FDC was performed similar to RT‐DC as described elsewhere.^[^ ^30^ ^]^ Briefly, cells were flushed through a constriction in a microfluidic chip at a flowrate of 0.06 µL s^−1^. A laser sheet was projected into the middle of the channel. When cells passed through the sheet, three lasers (488, 561, and 640 nm) excited the fluorescence signal and the fluorescence intensity was measured by dedicated detectors; resulting in three 1D‐fluorescence traces for each cell. Here, the maximum peak‐height of the fluorescence traces was used to quantify whether the corresponding cell was expressing a particular fluorescent marker. Since brightfield image and fluorescence acquisition were synchronized, the fluorescence information could be used as ground truth to label each image. RT‐FDC technology and all consumables are commercially available (Zellmechanik Dresden GmbH).

All studies complied with the Declaration of Helsinki and involved written informed consent from all participants. Donors were recruited at the University Medical Centre Carl Gustav Carus Dresden and ethics for experiments with human blood were approved by the ethics committee of the Technische Universität Dresden (EK89032013, EK458102015).

##### Statistical Analysis

All bar plots were expressed as the mean ± standard deviation (S.D.). Accuracy was calculated by dividing the total number of events by the number of correctly classified events. All statistical analyses were performed using Python 3.5.9.

## Conflict of Interest

The authors declare no conflict of interest.

## Author Contributions

M.H. and M.K. conceived the project, elected the experiments, and conducted training of NN in AID. M.H. developed the software AIDeveloper. M.H, M.K., A.J., and T.K performed experiments. M.K. conceived and performed the RT‐FDC experiment for B and T‐cells discrimination as well as the corresponding CNN training in AID. M.K. visualized the data and prepared all figures. M.H., M.K., S.A., and D.S. co‐wrote the manuscript. All authors reviewed the manuscript.

## Supporting information

Supporting Information

Supporting Information

Supporting Information

Supporting Information

Supporting Information

## Data Availability

All data used for training, validation, and testing, the final models, as well as the meta‐files to reproduce training procedure are publicly available at https://doi.org/10.5281/zenodo.3967000.
