# Supplementary material for: AIDeveloper: Deep Learning Image Classification in Life Science and Beyond
Source: Adv Sci (Weinh). 2021 Mar 18;8(11):2003743. doi: 10.1002/advs.202003743 (PMC8188199; doi:10.1002/advs.202003743)
Supplement: Supplementary file 1 — Supporting Information [file ADVS-8-2003743-s002.pdf]

WILEY-VCH

© 2021 Wiley-VCH GmbH

ADVANCED SCIENCE
Open Access

Supporting Information

for *Adv. Sci.*, DOI: 10.1002/advs.202003743

AIDeveloper: deep learning image classification in life science and beyond

Martin Kräter, Shada Abuhattum, Despina Soteriou, Angela Jacobi, Thomas Krüger, Jochen Guck, Maik Herbig**

Supporting Information

AIDeveloper: deep learning image classification in life science and beyond

Authors: Martin Kräter, Shada Abuhattum, Despina Soteriou, Angela Jacobi, Thomas Krüger, Jochen Guck, Maik Herbig

Supplementary figures and legends

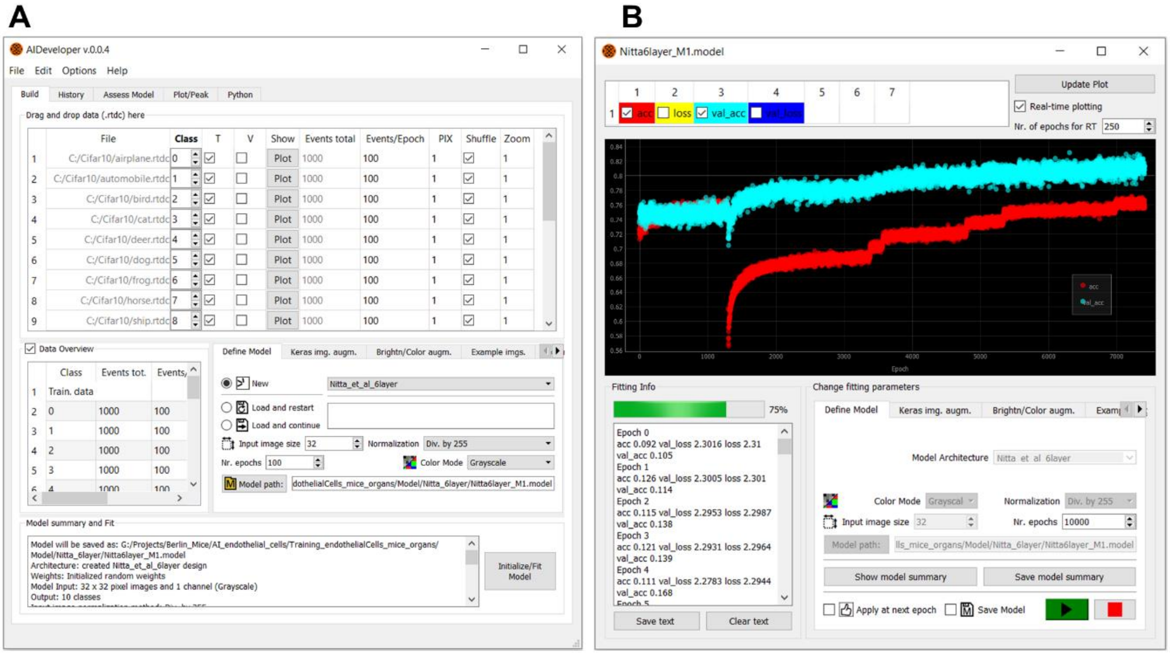

Figure S1: Screenshots of the graphical user interface (GUI) of AID

Intuitive elements allow the user to load data, select a neural net, and set hyper-parameters. (**A**) Main user interface of AID which shows a table of the loaded data and allows the user to define classes and which dataset belongs to training and validation set. Definition of the model is performed by choosing an architecture and input image size in corresponding GUI elements. (**B**) Real-time visualization of the progress during training of a specific model showing accuracy (red) and validation accuracy (cyan).

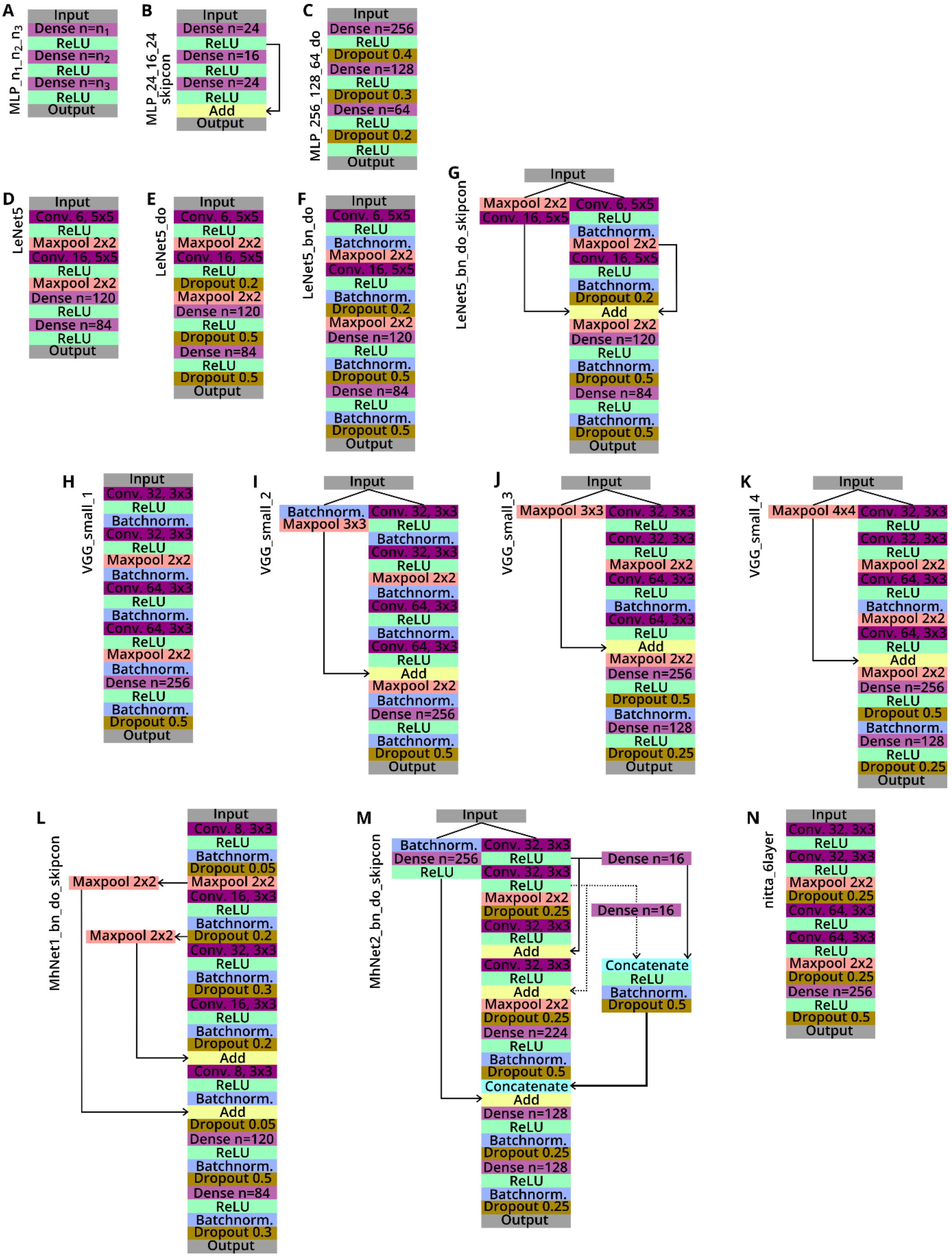

Figure S2: Sketches of neural nets (NN) that are implemented in AIDeveloper.

Each coloured box represents an individual layer of the network. Input layers in AID currently only accept squared images with either one (grayscale) or three channels (RGB

image). The output layer always consists of a fully connected layer including as many nodes as different output classes and a softmax activation layer [1]. Fully connected layers are abbreviated ’Dense’ and the number of nodes is given. In all shown NNs, rectified linear units (ReLU) are used as activation function. Convolutional layers are abbreviated ‘Conv.’ and the number of convolutional filters and the filter-size is given. Dropout layers are shown including their respective dropout rate. Downsampling is achieved using maxpooling layers and the pooling size is shown. Arrows indicate skip-connections, which bypass the main thread of the NN and either add or concatenate their data back to the NN in a subsequent layer.

A

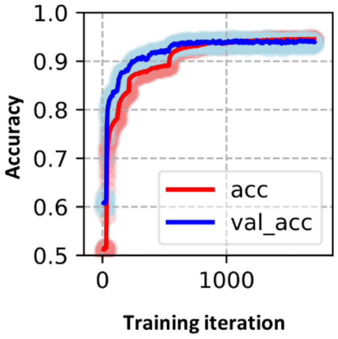

B

	Top	Trousers	Pullover	Dress	Coat	Sandal	Shirt	Sneaker	Bag	Boot
Top	904	0	13	13	1	0	67	0	2	0
Trousers	1	993	0	4	1	0	1	0	0	0
Pullover	13	0	906	10	36	0	34	0	1	0
Dress	9	1	2	967	14	0	7	0	0	0
Coat	0	0	27	20	908	0	44	0	1	0
Sandal	0	0	0	0	0	989	0	7	0	4
Shirt	95	1	41	28	36	0	797	0	2	0
Sneaker	0	0	0	0	0	12	0	958	0	30
Bag	3	0	0	1	3	1	3	0	989	0
Boot	0	0	0	0	0	1	0	27	0	972

Predicted label (columns); True label (rows)

Figure S3: Transfer learning approach for Fashion-MNIST

(**A**) The image shows a training process for the classification of fashion items from the Fashion-MNIST dataset. Here, transfer learning was applied by re-using a model (CNN_{gray}) previously trained on CIFAR-10. The red line (acc) shows the rolling median of the accuracy (window size = 10 training iterations) and light red indicates the accuracy of individual epochs. The blue line (val_acc) shows the corresponding rolling median of the validation accuracy and light blue indicates the validation accuracy of individual epochs. The model with the highest validation accuracy (95.1%) was applied to the testing set resulting in a testing accuracy of 93.8%. (**B**) The confusion matrix shows the performance of the final model on the testing set. The model appears to have most difficulties in distinguishing "Top" and "Shirt" (highlighted in orange).

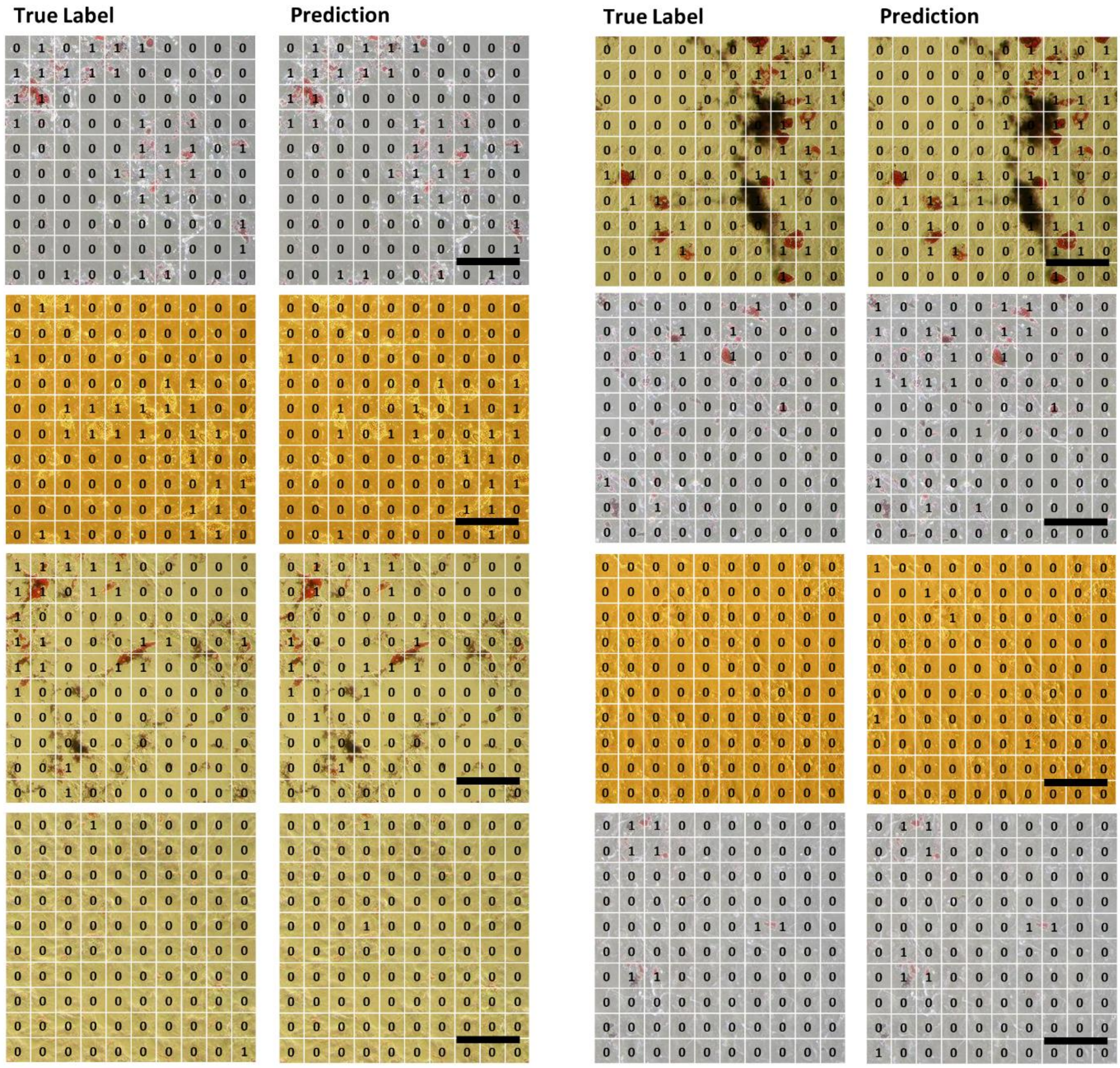

Figure S4: Adipocyte detection in validation images

The micrographs of MSC layers induced to differentiate into the adipogenic direction highlight the image colour and Oil Red O staining variability. Regions in the image containing differentiated adipocytes were marked by an expert (True label, left column). The trained neural net was applied to predict those regions (Predicted label, right column). Scale bars = 50 µm.

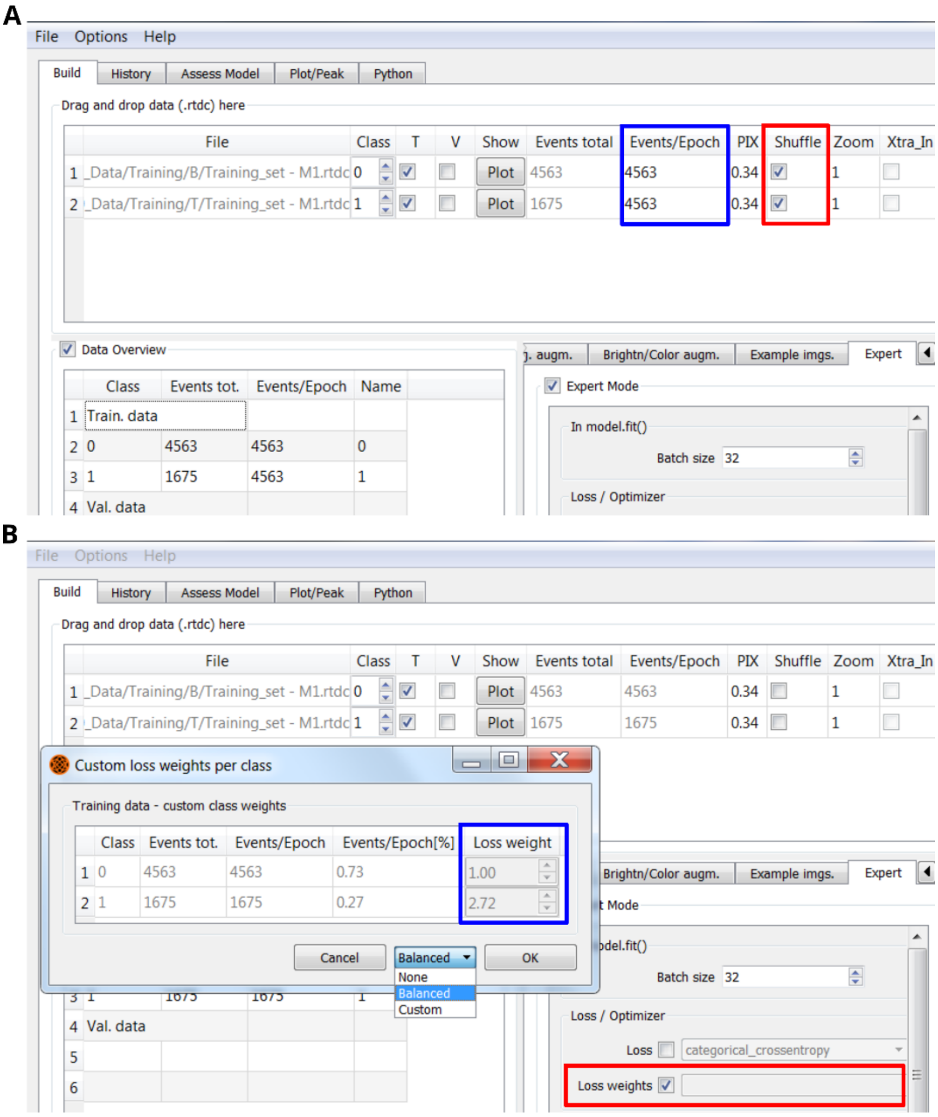

Figure S5: Methods for dealing with class imbalance

(**A**) Balancing a dataset using random sampling: the screenshot shows a dataset where class 0 and 1 contain 4563, and 1675 images, respectively. By selecting the option Shuffle (indicated by red rectangle), the user can choose how many images should be randomly sampled in each training iteration. In the example given, 4563 images are sampled from both files (indicated by blue rectangle), resulting in a balanced dataset.

(**B**) Balancing a dataset using loss weights: the screenshot shows the same dataset as in (A) but “Shuffle” is switched off. As a result, all images of each file are used. Upon activating “Loss weights” (indicated by red rectangle), a popup window allows the user to define custom loss weights. Alternatively, when selecting “Balanced” in the dropdown menu, AIDeveloper determines the loss weight factors that would correspond to a balanced loss. In the example given, class 0 comprehends approximately 2.7 times more image compared to class 0. Therefore, class 1 gets a loss weight of 2.7 in order to reach a balanced loss.

Supplementary tables

Table S1: Python libraries used

Name	Version	Build Channel
absl-py	0.9.0	<pip>
altgraph	0.17	<pip>
astor	0.8.1	<pip>
certifi	2018.8.24	py35_1
dclab	0.22.1	<pip>
et-xmlfile	1.0.1	<pip>
ffmpeg	1.4	<pip>
future	0.18.2	<pip>
gast	0.3.3	<pip>
grpcio	1.28.1	<pip>
h5py	2.10.0	<pip>
imageio	2.4.1	<pip>
intel-openmp	2019	<pip>
jdcal	1.4.1	<pip>
Keras	2.2.4	<pip>
Keras-Applications	1.0.8	<pip>
Keras-Preprocessing	1.1.0	<pip>
keras2onnx	1.4.0	<pip>
libopencv	0.0.1	<pip>
Markdown	3.2.1	<pip>
mkl	2019	<pip>
numpy	1.16.4	<pip>
onnx	1.6.0	<pip>
onnxconverter-common	1.6.1	<pip>
opencv-contrib-python-headless	4.1.1.26	<pip>
openpyxl	2.5.6	<pip>
pandas	0.24.0	<pip>
pefile	2019.4.18	<pip>
Pillow	5.4.1	<pip>
pip	10.0.1	py35_0
protobuf	3.11.3	<pip>
psutil	5.4.7	<pip>
PyQt5	5.9.2	<pip>
pyqtgraph	0.11.0rc0	<pip>
python	3.5.6	he025d50_0
python-dateutil	2.8.1	<pip>
pytz	2020.1	<pip>
pywin32-ctypes	0.2.0	<pip>
PyYAML	5.3.1	<pip>

scikit-learn	0.20.0	<pip>
scipy	1.4.1	<pip>
setuptools	46.1.3	<pip>
sip	4.19.8	<pip>
six	1.14.0	<pip>
tensorboard	1.12.2	<pip>
tensorflow-gpu	1.12.3	<pip>
termcolor	1.1.0	<pip>
tf2onnx	1.4.1	<pip>
typing	3.7.4.1	<pip>
typing-extensions	3.7.4.2	<pip>
vc	14.1	h0510ff6_4
vs2015_runtime	14.16.27012	hf0eaf9b_1
Werkzeug	1.0.1	<pip>
wheel	0.31.1	py35_0
wincertstore	0.2	py35hfebbdb8_0
xlrd	1.1.0	<pip>

	AID	Intellesis	Cell Profiler 3.0	deepImageJ	DIGITS	DLS	ilastik	KNIME	Weka
Open source	Yes	No	Yes	Yes	Yes	No	Yes	Yes	Yes
Deep learning out-of-the-box	Yes	Yes	No	Plugin	No	Yes	No	No	Plugin
GPU support out-of-the-box	Yes*	Yes	No	No	No	$	No	No	No
Multi-GPU support out-of-the-box	Yes*	No	No	No	No	$	No	No	No
Apply pre-trained model	Yes	Yes	Yes	Yes	Yes	Yes	Yes	Yes	Yes
Classification	Yes	No	No	No	Yes	Yes	No	Yes	Yes
Segmentation	No	Yes	Yes	Yes	Yes	Yes	Yes	Yes	Yes
Design DNN in UI	No	No	No	No	No	Yes	No	Yes	Yes
Train model	Yes	No	No	No	Yes	Yes	No	Yes	Yes
Transfer learning	Yes	No	No	No	Yes	Yes	No	Yes	No
Pre-trained models for transfer learning	Yes	No	No	No	Yes	Yes	No	Yes	No
Save model to Keras format	Yes	No	No	No	No	Yes	No	Yes	No
Save model to TensorFlow format	Yes	No	No	No	Yes	Yes	No	Yes	No
Save model to ONNX format	Yes	No	No	No	No	No	No	No	No
Save model to CoreML format	Yes	No	No	No	No	No	No	No	No
Offline execution	Yes	Yes	Yes	Yes	Yes	$	Yes	Yes	Yes
Image augmentation	Yes	No	No	No	Yes	Yes	No	No	No
Real-time feedback during training	Yes	No	No	No	Yes	Yes	No	Yes	No
Visualization of training data	Yes	No	No	No	No	Without augmentation	No	Yes	No
Parameter adjustment during training	Yes	No	No	No	No	No	No	No	No

Table S2: Comparison of software packages

The table compares multiple software tools for image analysis and deep learning with respect to the following features:

Open source: Is the full source code of the software freely available?

Deep learning out-of-the-box: Does the software include deep learning features by default?

GPU support out-of-the-box: Does the software allow for GPU computing by default?

Multi-GPU support out-of-the-box: Does the software support training of DNNs on multiple GPUs by default?

Apply pre-trained model: Does the software allow to load models to predict new data?

Classification: Does the software allow to train DNNs for classification tasks?

Segmentation: Does the software allow to train DNNs for image segmentation tasks?

Design DNN in UI: Does the software provide tools to design new DNNs within a user interface?

Train model: Is the software capable to train new models?

Transfer learning: Is the software equipped with options that allow to perform transfer learning. That requires loading of other models to continue training (on new or other data). Furthermore, there need to be options to keep certain layers non-trainable as for successful transfer learning typically, only the last layers are optimized.

Pre-trained models for transfer learning: Does the software provide pre-trained models, which can be used for transfer learning (e.g. models trained on ImageNet)?

Save model to Keras format: Does the software allow to export models to Keras format? Keras is a popular deep learning framework and saving in that format would allow to use the model outside the software.

Save model to TensorFlow format: Does the software allow to export models to TensorFlow format?

Save model to ONNX format: Does the software allow to export models to ONNX format?

Save model to CoreML format: Does the software allow to export models to CoreML format?

Offline execution: Can the software run autonomously, without internet connection? This feature might be important when data protection needs to be granted (e.g. for clinical data).

Image augmentation: Does the software provide options to augment the dataset (e.g. by applying random rotations or brightness changes to the images)

Real-time feedback during training: Is there a visualization of model metrics (e.g. accuracy) available during the training process in real-time?

Visualization of training data: Does the software allow to visualize the training image data?

Parameter adjustment during training: Does the software allow to adjust model hyper-parameters (e.g. learning rate, image augmentation parameters) during the training process?

(*) Windows only

Table S3: Comparison of models available in AIDeveloper

Model name	Valid input image sizes I	Valid number of channels	Conv. layer	Drop out layer	Batch norm layer	Skip conne ction	Pre-trained	Nr. of parameters for I=32	Nr. of parameters for I=48	Nr. of parameters for I=75	Nr. of parameters for I=224	Nr. of parameters for I=331
MLP_24_16_24_skipcon	≥1	1,3	No	No	No	Yes	No	74,810	166,970	406,082	3,613,754	7,889,474
MLP_256_128_64_do	≥1	1,3	No	Yes	No	No	No	828,490	1,811,530	4,362,058	38,577,226	84,184,906
LeNet5	≥16	1,3	Yes	No	No	No	No	62,006	169,526	446,006	5,407,286	11,996,726
LeNet5_do	≥16	1,3	Yes	Yes	No	No	No	62,006	169,526	446,006	5,407,286	11,996,726
LeNet5_bn_do	≥16	1,3	Yes	Yes	Yes	No	No	62,910	170,430	446,910	5,408,190	11,997,630
LeNet5_bn_do_skipcon	≥16	1,3	Yes	Yes	Yes	Yes	No	137,966	291,566	637,166	6,036,206	12,925,166
VGG_small_1	≥16	1,3	Yes	Yes	Yes	No	No	485,930	1,417,770	3,813,930	46,811,690	103,920,170
VGG_small_2	14≤I≤37	1,3	Yes	Yes	Yes	Yes	No	486,198	-	-	-	-
VGG_small_3	14≤I≤37	1,3	Yes	Yes	Yes	Yes	No	511,146	-	-	-	-
VGG_small_4	≥8	1,3	Yes	Yes	Yes	Yes	No	363,690	691,370	1,428,650	12,946,602	27,643,050
Nitta_et_al_6layer	≥16	1,3	Yes	Yes	No	No	No	477,994	1,395,498	3,754,794	46,091,050	102,320,938
Nitta_et_al_8layer	≥36	1,3	Yes	Yes	No	No	No	-	173,610	345,642	4,859,434	11,355,690
MhNet1_bn_do_skipcon	≥4	1,3	Yes	Yes	Yes	Yes	No	85,526	162,326	335,126	3,034,646	6,479,126
MhNet2_bn_do_skipcon	≥2	1,3	Yes	Yes	Yes	Yes	No	3,745,686	8,333,206	19,968,662	179,906,454	391,557,782
pretrained_squeezenet	≥48	3	Yes	Yes	Yes	No	ImgNt	-	1,907,530	3,218,250	45,161,290	95,492,938
pretrained_mobilenet	≥32	3	Yes	Yes	Yes	No	ImgNt	7,444,682	7,444,682	13,736,138	108,107,978	215,062,730
pretrained_mobilenet_v2	≥32	3	Yes	Yes	Yes	Yes	ImgNt	6,998,090	14,862,410	27,969,610	132,827,210	321,570,890
pretrained_nasnetmobile	224	3	Yes	Yes	Yes	Yes	ImgNt	-	-	-	112,360,094	-
pretrained_nasnetlarge	331	3	Yes	Yes	Yes	Yes	ImgNt	-	-	-	-	1,086,197,340
pretrained_densenet	≥32	3	Yes	Yes	Yes	Yes	ImgNt	11,253,322	11,253,322	17,544,778	111,916,618	218,871,370
pretrained_vgg16	≥48	3	Yes	Yes	Yes	No	ImgNt	17,881,930	17,881,930	21,027,658	68,213,578	121,690,954
pretrained_vgg19	≥48	3	Yes	Yes	Yes	No	ImgNt	23,191,626	23,191,626	26,337,354	73,523,274	127,000,650
pretrained_inception_v3	≥75	3	Yes	Yes	Yes	Yes	ImgNt	-	-	28,115,754	28,115,754	28,115,754
pretrained_xception	≥71	3	Yes	Yes	Yes	Yes	ImgNt	-	-	60,728,882	228,501,042	530,490,930
pretrained_resnet50	≥32	3	Yes	Yes	Yes	Yes	ImgNt	29,900,682	42,483,594	63,455,114	231,227,274	533,217,162
pretrained_resnet101	≥32	3	Yes	Yes	Yes	Yes	ImgNt	48,971,146	61,554,058	82,525,578	250,297,738	552,287,626
pretrained_resnet152	≥32	3	Yes	Yes	Yes	Yes	ImgNt	64,683,914	77,266,826	98,238,346	266,010,506	568,000,394
pretrained_resnet50_v2	≥32	3	Yes	Yes	Yes	Yes	ImgNt	29,877,770	42,460,682	63,432,202	231,204,362	533,194,250
pretrained_resnet101_v2	≥32	3	Yes	Yes	Yes	Yes	ImgNt	48,939,530	61,522,442	82,493,962	250,266,122	552,256,010
pretrained_resnet152_v2	≥32	3	Yes	Yes	Yes	Yes	ImgNt	64,644,618	77,227,530	98,199,050	265,971,210	567,961,098
pretrained_resnext50	≥32	3	Yes	Yes	Yes	Yes	ImgNt	29,361,098	41,944,010	62,915,530	230,687,690	532,677,578
pretrained_resnext101	≥32	3	Yes	Yes	Yes	Yes	ImgNt	48,579,530	61,162,442	82,133,962	249,906,122	551,896,010

The table shows the models available in AIDeveloper and corresponding properties. The model properties assessed by the table are as followed.

Model name: the name to identify the model in AIDeveloper.

Valid input image sizes I: the valid input image size I in pixels.

Valid number of channels: the valid number of image channels. Grayscale and RGB images correspond to 1, and 3 channels, respectively.

Conv. layer: specification if the model contains at least one convolutional layer.

Dropout layer: specification if the model contains at least one dropout layer.

Batch norm layer: specification if the model contains at least one batch norm layer.

Skip connection: specification if the model contains at least one skip connection.

Pre-trained: specification if the model was pre-trained on ImageNet (ImgNt). Pre-trained models are obtained from https://storage.googleapis.com/tensorflow/keras-applications/.

Nr. of parameters: the total number of parameters of the model if the number of channels is 3 (RGB), the number of output nodes is 10, and the input image size is 32, 48, 75, 224, or 331, respectively.

Supplementary videos

Video S1: Basic usage of AIDeveloper. Video showing how to load data into AID and start a training process: https://youtu.be/dvFiSRnwoto

Video S2: Editing neural nets in AIDeveloper. Video showing how to how edit the model_zoo.py to modify and add neural nets used in AID: https://youtu.be/XboH-YsG6LA

Video S3: Fashion-MNIST. Video showing how to use transfer learning in AID**:** https://youtu.be/NWhv4PF0C4g

Video S4: Creating a standalone executable of AIDeveloper on MAC. Video showing how to set up a Python environment for AID and freezing AID using PyInstaller on Mac: https://youtu.be/Ofd_8YBm4xI
